# A critical review of the relationship between type 1 diabetes mellitus, inhibition, and behavioral management

**DOI:** 10.3389/fcdhc.2022.1080415

**Published:** 2023-02-01

**Authors:** Neville Dean Robertson, Elmari Deacon, Karel Botha

**Affiliations:** ^1^ School of Psychosocial Health, Community Psychosocial Research (COMPRES), North-West University, Potchefstroom, South Africa; ^2^ OPTENTIA, North-West University, Vanderbijlpark, South Africa

**Keywords:** type 1 diabetes mellitus, inhibition or inhibitory control, behavior management, self-management, critical review

## Abstract

Type 1 diabetes mellitus (T1DM) is a chronic and lifelong condition that requires adequate behavior management in order to meet desired health outcomes. The effects of T1DM on the neurocognitive functioning of affected individuals raise concerns about how the disease may influence executive functioning. Inhibition is a core component of executive functioning, and plays a vital role in self-regulation and the restriction of impulsive behaviors. Inhibition may thus play a vital role in the behavior management of people with T1DM. The aim of this study was to identify current gaps in existing knowledge regarding the relationship between T1DM, inhibition, and behavior management. This study employed a critical review design to analyze and synthesize the current scientific literature. Twelve studies were identified through an appraisal process, and the data extracted were thematically analyzed and integrated. The findings of this study indicate that a possible cycle arises between these three constructs, in which T1DM affects inhibition, inhibition affects behavior management, and poor behavior management affects inhibition. It is recommended that future research should focus more specifically on this relationship.

## Highlights

- The relationship between type 1 diabetes mellitus (T1DM), inhibition, and behavioral management remains under-researched.
- Twelve studies were identified through an appraisal process, and these were thematically analyzed and integrated.
- A vicious cycle arises as the results indicate that there is an interdependent relationship between T1DM, inhibition, and behavioral management.

## Introduction

The current scientific literature explores the relationship between type 1 diabetes mellitus (T1DM) and executive functioning (EF); however, research exploring T1DM and inhibition specifically as they relate to behavior management is limited. Inhibition is important in the control and adjustment of behavior, emotion, and cognition, and, considering the complex demands of managing a condition such as T1DM, it is an important function to consider. T1DM is considered a severe, chronic, and lifelong condition, and commonly manifests as the presence of high blood sugar levels (1). It is an autoimmune condition resulting in the destruction of pancreatic β-cells, which produce the hormone insulin, ultimately contributing to varying glycemia (2–4). Gregory et al. (3) describe T1DM as glucose disequilibrium that leads to insulin deficiency, hypoglycemia, or hyperglycemia, thereby contributing to various adverse ramifications. Hypoglycemia occurs when the blood sugar levels of an individual are lower than normal, whereas hyperglycemia refers to blood sugar levels that are too high. Poor glycemic control in individuals living with T1DM may affect both the brain structure and cognitive functioning of an individual.

T1DM is severe and leads to various complications, such as neuropathy, loss of consciousness, seizures, cardiovascular difficulties, kidney failure, blindness, and neurocognitive difficulties (5–12). The brain is dependent on glucose availability, which is crucial for its functioning and development (13). Brain structure and function have been found to differ in individuals with and without T1DM (5–7, 12, 14–16). The severe and continuous fluctuation of glycemia may be detrimental to the brain and neurological functioning (7). For example, severe increases and decreases in blood sugar levels have been associated with adversely affecting various mental activities and neurological development, and contributing to cognitive dysfunction (17).

One of the neurocognitive difficulties identified through research is difficulty in executive functioning (8, 13–15, 18–27). Executive functioning is considered an umbrella term that encompasses a diversity of cognitive processes (28), including working memory, cognitive flexibility, attention, and inhibition. Miller et al. (22) state that executive functioning and its related processes are of clinical relevance in T1DM because of the behavioral management that is required in the multifaceted demands of various executive functioning competencies, such as inhibition. Inhibition, also known as inhibitory control, is a central construct in executive functioning and related processes, and difficulties in the area of inhibition may affect other aspects of brain function, such as updating, shifting, problem-solving, attention, planning, and control (29). When one examines the function of inhibition and the requirements of T1DM management, inhibition may be of considerable importance in the management of diabetes, and in turn may be adversely affected by T1DM (13).

Inhibition, according to Chung et al. (30) and Goldmann-Rakic et al. (31), enables individuals to reject automatic tendencies. Caruso et al. (19) offer a more specific definition, describing inhibition as the ability to utilize interference control and/or response inhibition to control emotions, thoughts, behavior, and attention (19). Inhibition enables an individual to exert self-control by resisting temptations and acting impulsively (32). An individual who can exert self-control is better able to resist various temptations and impulses, such as the urge to overindulge when eating, to impulsively respond, or to behave in a manner that may cause harm, and to exercise self-discipline. This may be of extreme importance when considering diabetes behavior management, as it may assist individuals with T1DM to adhere to diabetes care plans, for example by resisting the urge to eat when glycemia is high and administering insulin at the correct times.

Swanson and Maltinsky (33) assert that many individuals struggle to maintain control in the management of T1DM. The management of T1DM involves various complex tasks regarding control and adjustments to one’s lifestyle. Key self-management behaviors that may promote healthy outcomes in individuals with T1DM include (but are not limited to) regular self-monitoring of blood glucose levels, following strict diet recommendations, adhering to diabetes care plans, being prepared for unexpected events, and resisting certain urges and impulses (33–35).

## Problem statement

Thus far, the literature has predominantly placed the focus on the umbrella term, executive functioning, as it relates to T1DM and the behavioral management of people with T1DM. Moreover, the existing literature has mainly been concerned with a one-directional relationship: the influence of executive functioning on T1DM management. Limited scientific literature has placed the focus on inhibition, T1DM, and behavioral management. More specifically, research that examines the bidirectional relationship between inhibition, T1DM, and behavioral management is limited.

The findings of this study may provide valuable information and identify current gaps within existing knowledge to enable future research to adopt a more specific focus. Therefore, the proposed study aimed to answer the following research question: *According to the scientific literature, what conclusions can be drawn regarding inhibition, T1DM, and the management of T1DM?*

## Method

### Research design

A critical review provided the primary researcher with the necessary framework to meet the study’s aims of evaluating and integrating the existing body of knowledge to provide an in-depth and holistic perspective on the phenomena (36). It is of the utmost importance to note that a critical review moves beyond mere description and involves a “degree of analysis and conceptual innovation” (37, p.93). Therefore, because the purpose of the proposed study is to provide an integrated perspective through the examination, evaluation, and analysis of the current scientific literature, a critical review approach was deemed the most suitable.

### Research approach

EBSCO*host* (EBSCO Information Services, Ipswich, MA, USA) [including but not limited to International Scientific Indexing (ISI, Jumeirah Village, United Arab Emirates), MEDLINE^®^ (National Library of Medicine, Bethesda, MD, USA), and Scopus^®^ (Elsevier, Amsterdam, the Netherlands)], ScienceDirect^®^ (Elsevier), Google Scholar (Google Inc., Mountain View, CA, USA), Academic Search Premier (EBSCO Information Services), SocINDEX with Full Text (EBSCO Information Services), PsycArticles^®^ (American Psychological Association, Washington, DC, USA), PsycInfo^®^ (American Psychological Association), and JSTOR were included in the search to obtain all relevant, credible, rigorous, and published scientific literature. The primary reviewer (first author) performed an independent search for scientific literature, which was continuously monitored by the secondary reviewer (second author). The secondary reviewer acted as a co-analyst regarding the extracted data.

**Figure 1:**
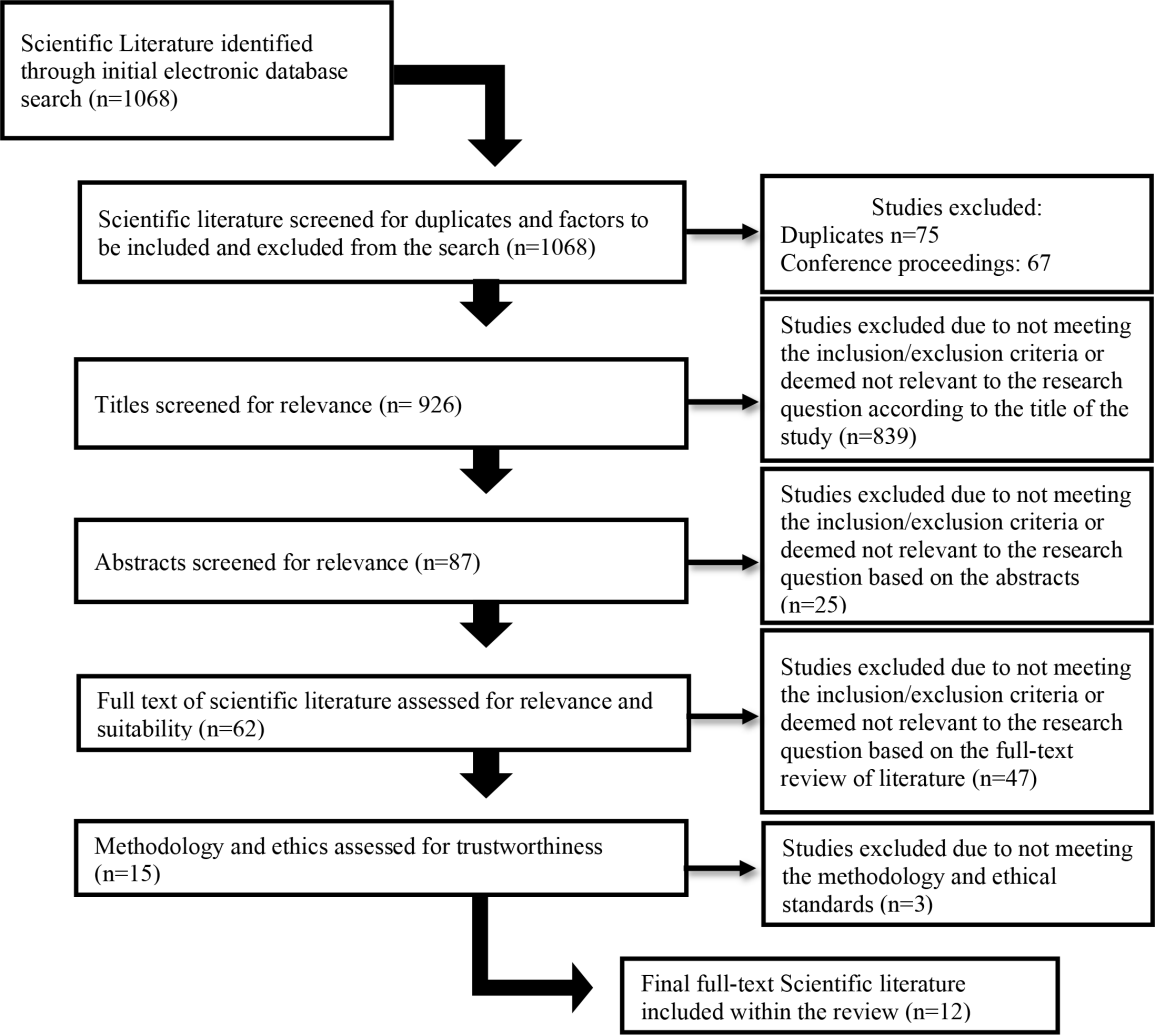
Flow chart of critical literature review and inclusion or exclusion thereof.

Certain keywords were utilized in the search of the above-mentioned databases: Inhibition OR Inhibitory control OR Cognitive Inhibition OR Response Inhibition OR Interreference Control AND Type 1 Diabetes OR Type 1 Diabetes Mellitus OR T1D OR T1DM OR ‘Insulin dependent Diabetes OR Juvenile Diabetes AND Self-management OR ‘Behavioral Management’ OR ‘Diabetes care plans’ AND ‘Executive functioning OR Executive Function OR Executive Dysfunction OR Cognitive Dysfunction. Studies were excluded if they focused only on T1DM, inhibition, or behavior management alone. No specified age ranges were used in the search, as studies may provide valuable information regarding differences among age groups. Furthermore, studies were included only if they adhered to ethical and methodological principles. **Figure 1** depicts the process through which the search was conducted and how scientific literature was included or excluded.

A total of 1,068 articles were identified during the initial search. After excluding duplicates and conference proceedings, 926 remained. In the subsequent appraisal process, article titles and abstracts were reviewed in accordance with the inclusion and exclusion criteria of the study. The inclusion criteria for the study were scientific literature that focuses on (1) inhibition, (2) T1DM, and/or (3) behavior management. The exclusion criteria of the study were scientific literature that (1) focuses on only one of the above-mentioned criteria and (2) focuses on type 2 diabetes. The above-mentioned criteria were utilized when evaluating the relevance of the scientific literature identified. Based on the titles and abstracts of the scientific literature, 62 articles progressed to the next stage, in which the full text was reviewed in order to determine the relevance of the article to our research. A total of 15 remained, three of which did not meet the criteria for ethical and methodological standards. Therefore, 12 articles were included in this review. The primary and secondary reviewers consulted during the process, and consensus was reached regarding the inclusion and exclusion of articles. **Table 1** provides an outline of each of the articles identified and included in this study.

**Table 1:** Data extraction table.

Author(s), year of publication, and title	Overview and details of the study	Sample	Results and findings	Other
Broadley et al. (13) *A Systematic Review and Meta-analysis of Executive Function Performance in Type 1 Diabetes Mellitus*	*Aim:* (1) To examine the current evidence for executive functioning performance differences between groups with T1DM and non-diabetic control groups during adolescence and early adulthood (2) To explore the relationships between EF and diabetes-related risk factors *Design*: A systematic review of the scientific literature *Method:* PRISMA guidelines and a meta-analysis of a subset of the articles	Electronic database searches for published and unpublished literature yielded a final set of 26 articles after application of inclusion and exclusion criteria	16 out of the 16 scientific articles included in the study indicated significantly lower EF with T1DM. Of relevance to this study was that the subset of inhibition was significantly lower in individuals living with T1DM	*Conclusion:* T1DM and its associated risk factors are related to subtle impairments across the inhibition, working memory, and set-shifting domains of EF. Lower EF may be a key factor contributing to behavioral and clinical problems experienced by individuals with T1DM
Caruso et al. (19) *Sleep, Executive Functioning and Behavior in Children and Adolescents with Type 1 Diabetes*	*Aim*: To examine sleep and neurocognitive and behavioral functioning in children and adolescents with T1DM compared with control group participants, and to test whether or not sleep quality mediates the relationship between diabetes and neurocognitive and behavioral deficits. *Measures:* Survey consisting of the Sleep Disturbances Scale for Children/Behavior Rating Inventory of Executive Functions/Behavior Assessment System for Children-2/diabetic and demographic parameters were collated from medical records	Forty-nine children and adolescents with T1DM (recruited from a hospital clinic) and 36 control participants (age range 6–16 years)	Children with T1DM, compared with control group participants, reported a higher frequency of sleep problems and mild deficits in executive and behavioral functioning, such as inhibition	*Conclusion:* The neurocognitive and behavioral deficits in children with T1DM can be explained by the effect of T1DM on sleep and the resulting sleep disturbances
Duke et al. (20) *The Diabetes Related Executive Functioning Scale* *(DREFS): Pilot Results*	*Aim:* To develop and pilot test the Diabetes Related Executive Functioning Scale *Design:* Cross sectional design *Measures:* DREFS/BRIEF/BRIEF-SR/SA-DSMP/glycemic control	Fifty adolescents with T1DM and their caregivers were recruited from a regional pediatric diabetes center in the US Pacific Northwest	All relationships between subscales were significant at a *p*-value < 0.01, except for the relationship between mental flexibility and planning for youth self-report (*p* < 0.05), and between emotional regulation and organizing materials for the caregiver report (*p* < 0.05). Inhibition was significantly correlated with other subscales and HbA_1c_ levels	*Limitations:* The predictive, divergent, and incremental validities of the DREFS were not established in this study. Given these limitations, the results may not be generalizable to other settings or populations *Implications:* Using the DREFS in clinical settings promises to have important practical utility. DREFS scores can yield information about specific EF and related behavior problems that are clinically relevant and important to optimizing diabetes management
Foland-Ross et al. (14) *Executive Task-based Brain Function in Children with Type 1 Diabetes: An Observational Study*	*Aim*: To investigate activation patterns using functional magnetic resonance imaging as they performed an executive function paradigm, the go/no-go task *Design*: Multisite study framework *Measures*: Cognitive testing and blood glucose measurement/MRI acquisition/Go/no-go task design/behavioral data analyses	Ninety-three children with T1DM (mean age 11.5 ± 1.8 years; 45.2% female) and 57 non-diabetic (control) children (mean age 11.8 ± 1.5 years)	Equivalent performance on the go/no-go task between control and T1DM groups	*Conclusion:* Despite equivalent cognitive and behavioral functioning between groups, young children with T1DM exhibited increased activation in executive control regions
Graveling et al. (38) *Acute Hypoglycaemia Impairs Executive Cognitive Function in Adults with and without Type 1 Diabetes*	*Aim*: To examine the effect of acute hypoglycemia on executive function in adults with and without diabetes *Measures*: The National Adult Reading Test (NART)/Delis–Kaplan Executive Function (D-KEFS) Test suite/category switching/Twenty Questions Test/Tower test/Color–word Interference Test (Stroop)	Thirty-two adults with and without T1DM with no vascular complications or impaired awareness of hypoglycemia were studied	Executive functions (with one exception) were significantly impaired during hypoglycemia compared with euglycemia, lower test scores were recorded, with more time required for completion. Large Cohen *d*-values (0.8) suggest that hypoglycemia induces decrements in aspects of executive function	Executive cognitive function, which is necessary to carry out many everyday activities, is impaired during hypoglycemia in adults with and without T1DM
Hamburger et al. (39) *Performance-based and Questionnaire Measures of Executive Function in Adolescents with Type 1 Diabetes*	*Aim*: To examine EF in adolescents with T1DM *Design*: Quantitative descriptive design *Measures*: Updating/working memory/EF shifting/EF inhibition/BRIEF/self-care inventory/blood glucose monitoring/glycemic control	Data analyses included 65 adolescent (age range: 13–17 years)–parent dyads	None of the performance-based measures of EF was significantly associated with adherence or with HbA_1c_ level. Parent-reported problems with EF were associated with poorer adherence, and adolescents who scored in the impaired range of the Behavioral Regulation Index of EF had significantly poorer adherence	*Conclusion*: The findings suggest that parent-reported measures of EF may be more strongly linked to diabetes indicators than performance-based measures
Hanna et al. (40) *Association of Habits, Triggers, Glycemic Control, Routines, Stress* *and Impulse Control Among Emerging Adults with Type 1 Diabetes*	*Aim:* To examine associations among CBG habits, EAM habits, and glycemic control within the context of CBG triggers, daily routines, impulse control, and perceived daily stress, in emerging adults with T1DM *Design:* Multimethod design *Method*: Self-report and path analysis	A convenience sample of 100 emerging adults with T1DM was recruited from an outpatient diabetes care clinic for this age group	*Results:* Better glycemic control was positively and significantly associated with greater frequency of CBG and with good EAM habits. CBG habits were positively and significantly associated with CBG triggers and EAM habits. EAM habits were positively and significantly associated with daily routines	*Conclusion*: We suggest that interventional research targeting CBG and EAM habits and daily routines, to examine the impact on diabetes self-management and glycemic control, is undertaken
Łuczyński et al. (41) *The Empowerment of Adolescents with Type 1 Diabetes Is Associated with Their Executive Functions*	*Aim:* To determine the readiness for change of adolescent patients with T1DM as it related to clinical features and executive functioning *Design:* Cross-sectional study *Measures*: Diabetes Empowerment Scale/Behavioral Rating Inventory of Executive Functions—Self Report Version	Comparison group (*n* = 112) Adolescents aged 14–18 years (*n* = 147)	It was observed that adolescents with T1DM had a higher rate of abnormal results in EF tests than their peers without diabetes	*Conclusion*: It is proposed that individualized diabetes education is given to this group of patients based on the assessment of readiness to change and executive function
**Miller et al. (2013)** (22) *Changes in Executive Functioning and Self-Management in* *Adolescents With Type 1 Diabetes: A Growth Curve Analysis*	*Aim*: To investigate the relationship of changes in executive functioning to changes in diabetes self-management *Design*: Longitudinal design *Measures:* Behavior Rating Inventory of Executive Functioning/diabetes self-management profile/glycemic control/parent report on child autonomy	Inclusion criteria included diagnosis of type 1 diabetes for at least 1 year, aged 9–11 years, and absence of potential secondary causes of T1DM diagnosis (*n* = 240)	Youth-reported self-management decreased over time, whereas behavioral regulation increased. Changes in behavioral regulation significantly predicted the rate of change in Youth-reported self-management	*Conclusion*: Positive changes in behavioral regulation may enhance self-management of T1DM during the transition to adolescence
Ohmann et al. (42) *Cognitive Functions and Glycemic Control in Children and Adolescents with Type 1 Diabetes*	*Aim*: To compare the quality of GC and cognitive measures in adolescents with T1DM to find out if the quality of diabetes management is related to cognitive impairment *Method*: Control group experimental design *Measures*: Wechsler Intelligence Scale for Children—III (WISC-III) German version/Wisconsin Card Sorting Test/Interference [Stroop Color–Word Task/Trail Making Test Part A/Youth Self- Report/Child Behavior Checklist	Seventy adolescent patients with T1DM and 20 age-matched controls	Impaired EFs, mainly problems of concept formation (*p* = 0.038), cognitive flexibility (*p* = 0.011), and anticipation (*p* = 0.000), were found in the patients with T1DM	T1DM is associated with cognitive deficits in adolescents independent of the quality of metabolic control and the duration of the disease. These deficits are probably related to the disease, especially in patients with early-onset diabetes
Strachan et al. (43) *Recovery of Cognitive Function and Mood after Hypoglycaemia in Adults with Insulin Treated Diabetes*	*Aim:* To explore the time required for cognitive functions and mood to return to normal after an acute episode of severe hypoglycemia *Design*: Quantitative exploratory design *Measures:* Various standardized neuropsychological tests	Forty subjects with insulin-treated diabetes were studied, most of whom were attending the Diabetes Outpatient Department at the Royal Infirmary of Edinburgh for regular review (*n* = 20 experienced severe hypoglycemia; *n* = 20 did not experience severe hypoglycemia)	For most of the cognitive tests, no evidence of a “hangover” effect of the acute hypoglycemia on cognitive function was observed. Subjects who had chronically elevated levels of depression and anxiety persistently performed more poorly in several cognitive tests, such as the Digit Symbol Test and the Stroop Task	These results suggest that recovery from any acute cognitive decrement after severe hypoglycemia was complete by 1.5 days. The cognitive decrements and altered mood states noted in the hypoglycemic subjects may be persistent and may be a consequence of previous exposure to recurrent episodes of severe hypoglycemia
Rovet and Alvarez (44) *Attentional Functioning in Children and Adolescents with IDDM*	*Aim*: To determine whether or not specific attentional cognitive processes are disrupted in children and adolescents with T1DM. *Design*: Longitudinal design *Measures*: Wechsler Intelligence Scale for Children—Revised/Modified Matching Familiar Figures Test/Stroop Color Word Test/Trail Making Test/Wisconsin Card Sorting Test/Continuous Performance Test	One hundred and three children and adolescents with T1DM and 100 control participants (age range 9.3–18.3 years)	Those having had seizures demonstrated a lower verbal IQ and greater difficulty with select, focus, and inhibit attentional components, whereas sustain, suppress, and shift attentional components were unaffected. Correlation analyses showed that higher concurrent blood glucose levels were associated with a reduced ability to inhibit impulsive responses, whereas multiple regression analyses indicated that inhibition and focus were best predicted by age at onset and concurrent blood glucose level	In children and adolescents with T1DM, attention is poorer in several but not all aspects of attention; these aspects are affected by a history of seizures from hypoglycemia and higher ambient blood glucose levels at time of testing. These results suggest both organizational and activation effects of diabetes on specific subcomponents of attention in diabetes

BRIEF, Behavior Rating Inventory of Executive Function; BRIEF-SR, Behavior Rating Inventory of Executive Function, self-administered; CBG, checking blood glucose; DREFS, Diabetes Related Executive Functioning Scale; EAM, eating a meal; EF; executive function; GC, glycemic control; HbA_1c_, glycated hemoglobin; IQ, intelligence quotient; PRISMA, Preferred Reporting Items for Systematic Reviews and Meta-Analyses; SA-DSMP, Self-Administered Diabetes Self-Management Profile; T1DM, type 1 diabetes mellitus.

### Data analysis

The thematic analysis process, as put forth in Braun and Clarke (45), was followed. The process comprises the following steps: (1) become familiar with the data, (2) code, (3) search for themes, (4) review themes, (5) define and name themes, and (6) write up the report. The first phase involved reading and re-reading each of the scientific articles identified. The researcher was thus able to become familiar with each of the articles identified, more specifically the findings and interpretation thereof (46). Active note-taking was also implemented during the first step and facilitated the identification of prominent patterns that arose within the articles, which were in turn used to generate preliminary codes. The second step involved the development of possible codes, which were considered a representation of the most prominent patterns that arose and were utilized throughout the subsequent steps. Each of the preliminary codes was recorded in Microsoft Excel to ensure that an accurate account of the process was kept and no duplicates had been coded.

Once the second step was concluded, the third step commenced, in which the previously generated codes were utilized (46), and correlated with one another to develop possible themes. The identification of themes was subject to review to ensure that each theme was accurate, represented each of the scientific articles, and answered the research question of the study. After each theme had been identified, the primary reviewer named and defined each of the themes that contributed to the value of each theme. Further refinement of each theme occurred through identifying the essence of each theme. This also ensured that each of the themes would be distinguishable from another. After thoroughly establishing each theme, the fifth phase, writing up the findings, commenced (46), after which the final phase consisted of integrating and providing an in-depth discussion of the findings.

Phases 2 through 5 did not only occur once, but continuously, and in a bidirectional manner, and this enabled the primary reviewer to continuously analyze and adjust the themes. Writing up of the findings occurred in phase 6, and the report produced was clear, concise, and non-repetitive.

## Findings and discussion

Two themes with related subthemes emerged from the analysis of the scientific literature identified (1) the influence of T1DM on inhibition and (2) the influence of poor inhibition on the self-management of T1DM. The findings, in general, indicate that inhibition may play a vital role for individuals living with T1DM. The results also indicate that inhibition is negatively affected by T1DM and is, therefore, associated with poorer management of the condition. The majority of the scientific literature revealed a significant relationship between T1DM, inhibition, and the behavior management of individuals with T1DM. The findings are not definitive but do indicate that there is an urgent need for future research, especially when one considers the cycle of effects that arise from the findings.

### Theme 1: T1DM influence on inhibition

The first theme that arose pertains to the effect that T1DM may have on inhibition in individuals living with T1DM. This theme is, therefore, defined as the manner in which T1DM may have an effect on inhibition. Differences occurred in the literature as to the manner in which inhibition is affected by the T1DM condition.

#### Subtheme 1.1: Glycemia’s influence on inhibition

The most prominent and consistent finding across the literature was the correlation between high glycemia and poor inhibition. This theme is defined as the effect of glycemia on inhibitory performance. More specifically, the findings indicate that hypoglycemia and hyperglycemia have a significant impact on inhibition. For example, (13, p. 14) state that “… these results may suggest that complications of chronic hyperglycaemia, not HbA_1c_ alone, are related to inhibition difficulties in T1DM.” Therefore, these findings suggest that it is not merely a high level of glycemia that results in difficulties with inhibition, but the complications that arise because of hyperglycemia. Hyperglycemia may result in neuropathy (nerve damage) and microvascular complications, thereby affecting neurons within the brain (47). Broadley et al. (13) found that lower performance related to background retinopathy, a consequence of hyperglycemia, may also have an effect on inhibition, leading to lowered inhibition performance.

Lower inhibition performance was further correlated with hypoglycemia in Broadley et al. (13), Duke et al. (20), Graveling et al. (38), Ohmann et al. (42), Strachan et al. (43), and Rovet and Alvarez (44). These articles indicated that inhibition may be affected by the low blood sugar levels of an individual. For example, Strachan et al. (43) found that individuals who experienced hypoglycemia performed poorly on the Stroop task: “demonstrated that performance on the DS and Stroop Task was poorer in the hypo subjects at all three time points (*p* < 0.05)”. What was of interest in the study of Rovet and Alvarez (44) was that they found that individuals with a history of seizures due to hypoglycemia were reported to experience lower inhibition. An explanation for such a correlation was not provided and remains open for speculation. Povroznik et al. (48) state that a stroke can lead to reduced blood flow and glucose supply to the brain, resulting in impairments. A stroke can also lead to metabolic stress and the death of brain cells, which may offer an explanation for the long-term effects of stroke in affected individuals (48). Research conducted regarding strokes and inhibition has found that, post stroke, individuals often experience various inhibition difficulties (49–51).

It has, however, become evident that the experience of hypo- or hyperglycemia has an impact on the inhibition performance of an individual living with T1DM. Duke et al. (20), for example, report a significant correlation between glycemia and inhibition, and Rovet and Alvarez (44) specifically highlight that hyperglycemia has a significant effect on the inhibition performance of individuals. These findings substantiate the effect that hypo- and hyperglycemia may have on individuals living with T1DM. Possible explanations for this effect, however, are rarely found in the literature. One possible explanation is the brain’s dependence on a continuous and consistent supply of glucose, which is adversely affected by fluctuations in the supply of glucose caused by hypo- or hyperglycemia (13).

#### Subtheme 1.2: Age at onset

The second subtheme that emerged is that the age at which an individual begins to experience symptoms of T1DM influences their inhibition performance. Luczynski et al. (2019) found that inhibition was lower in adolescents with T1DM than in control group participants, whereas Rovet and Alvarez (44) found lower inhibition only in children. (13, p 16) found that lower inhibition was associated with early-onset diabetes (EOD): “Specifically, preliminary evidence suggests that EOD … may be associated with lower inhibition”. Another example of this subtheme is found in (13, p. 13): “Taken together, this evidence suggests that those with EOD (relative to late-onset diabetes (LOD)) may experience greater inhibition difficulties”. These findings are of importance when considering the neurodevelopmental level of children and adolescents. Inhibition and other executive functions have been found to be located within the prefrontal cortex of the brain (52). The prefrontal cortex, from a developmental perspective, only starts to develop in early childhood to early adolescence until fully developed in early adulthood (53). Thus, children and adolescents are not able to fully utilize the ability to inhibit responses or to exert interference control. Children and adolescents are subsequently more likely to engage in risk-taking behavior (54). Risk-taking behavior in children and adolescents living with T1DM may involve not checking glycemia, missing meals, or forgetting to administer insulin.

The lack of prefrontal cortex development in children and adolescents may offer an explanation as to why early-onset diabetes can have a significant effect on their inhibition performance. One may argue that because inhibition is not yet fully developed, performance will be lower, and despite the developmental evidence that children and adolescents will not have the full capacity to utilize inhibition: when compared to control group participants, inhibition was consistently lower in children and adolescents living with T1DM than in their control group peers.

#### Subtheme 1.3: General lower inhibition performance

One of the main subthemes that arose was that individuals living with T1DM were reported to have, in general, lowered inhibition. This theme is, therefore, simply defined as the impact of T1DM on inhibition performance, without reference to specific factors. The findings and discussion of the scientific literature failed to report what specific factor of T1DM led to lowered inhibition performance. The focus of these studies was to determine the relationship between executive functioning and T1DM. Due to inhibition being a subscale on the assessments and measurements used, authors briefly depicted the lowered inhibition scores when compared with control group participants. The results of these studies indicated that individuals with T1DM had lower inhibition scores when compared to control group participants. Despite the ambiguous nature of these results, this finding is an important factor in this critical review, as it points to the fact that aspects of T1DM contribute to lower inhibition. More importantly, it seems that an aspect of T1DM, such as glycemia, does not affect inhibition independently. It is rather multiple factors that are interacting with one another. The general impact further indicates that despite adequate control regarding T1DM, the effects on inhibition are still evident, as other factors also play a vital role (13). These other factors, however, remain unknown and, therefore, require more specific exploration. Foland-Ross et al. (14)Graveling et al. (38), and Łuczyński et al. (41) obtained similar findings, in that a small difference in inhibition was identified in those living with T1DM. Ohmann et al. (42) specifically highlight that a general impairment in inhibition was found in the Stroop Task results of participants living with T1DM.

#### Subtheme 1.4: Neurological effect of T1DM

During the analysis, two studies found that T1DM may have an impact on certain structures or brain regions that are responsible for inhibition. Caruso et al. (19) state that the brain regions that are affected by T1DM are the prefrontal cortex and the limbic system. The reason why these structures are affected, according to Caruso et al. (19), is that these brain regions have an increased density of insulin receptors. Therefore, these brain regions are sensitive to glycemia and, as a result, fluctuating glycemia may have an adverse effect on these regions. 44, p. 807) highlight that the dorsolateral prefrontal and motor cortices are related to the poorer inhibitory function of the individual, as diabetes adversely affects these structures within the brain: “… whereas response inhibition is thought to involve the dorsolateral prefrontal and motor cortices”. The adverse effects of T1DM on the brain in general, but also on the specific regions mentioned above, are corroborated by the findings of Russo et al. (55; Kern et al. (56); Page et al. (57)Mortby et al. (58), and Rosenthal et al. (59). These findings have clinical implications when one considers the fact that inhibition has started to develop in childhood and continues to develop in adolescence. According to Liu et al. (60), the development of inhibition relies on the functional maturation of the frontal lobe; prefrontal cortex changes that occur during development result in the development of inhibition (60). However, the literature indicates that T1DM affects the development of this brain region, thus influencing the development of inhibition. In the case of early-onset diabetes in children, fluctuating glycemia may have an adverse effect not only on the utilization of inhibition but also on the development thereof.

What is of interest to note is that Rovet and Alvarez (44), as briefly mentioned before, found that a history of seizures adversely affects the performance of inhibition. This finding was specific to seizures that were induced by hypoglycemia, “… whereas a history of hypoglycemia-induced seizures was associated with poorer inhibit“ (44, p. 807). This was of specific relevance in children: “… showed that children with a positive seizure history were less likely to inhibit a response before adequately processing information about the second stimulus…” (44, p. 807). As a result, these findings indicate that there is a direct association between the impact of T1DM on neurological development, structure, and function, and inhibition.

### 1 Theme 2: Low inhibition effect on self-management of type diabetes mellitus management

The first theme described the effect that aspects related to T1DM, such as age at onset and fluctuating glycemia, on inhibition. The cycle that arose out of the findings is continued in theme 2, which describes the effect of poor inhibition (because of T1DM) on the behavior management of individuals living with T1DM. Inhibition, as an executive function, may play an important role in the self-management behavior of an individual living with T1DM, because of its important role in controlling thoughts, emotions, and behavior (61). This theme and accompanying subthemes arose during the analysis and encompass the areas of T1DM management that are affected because an individual living with T1DM is more likely to experience poor inhibition.

#### Subtheme 2.1: Poor impulse control

The first subtheme can be defined as the difficulty among individuals living with T1DM of inhibiting, stopping, or controlling impulsive behaviors or decisions. During the analysis, it became evident that individuals who live with T1DM often find it difficult to control impulses, as there is a significant correlation with poor control over self-management behaviors, such as control of glycemia. Broadley et al. (13) highlight that one of the functional impairments that occurred in inhibition resulted in poor impulse control and at-risk behavior. These findings are in accordance with the research of (62), who state that lower impulse control may lead individuals to pursue gratification, regardless of goals. Furthermore, impulse control, specifically the management thereof, has often been associated with T1DM, and is most strongly correlated with the ability to control one’s impulses, leading to better health outcomes related to T1DM (20). Considering that inhibition is the ability to override planned or initiated response, it becomes more apparent why it plays such a significant role in controlling impulses in order to facilitate goal-directed behavior. Łuczyński et al. (41) note that inhibition plays an important role in the setting and achievement of goals as it relates to health outcomes. These findings of the relationship between inhibition and impulse control are corroborated by Bari and Robbins (63), (64), and Roberts et al. (65).

The study of Rovet and Alvarez (44) found that hyperglycemia contributes to poor impulse control. Therefore, an individual with poor glycemic control may have difficulty in inhibiting impulses (39). Hanna et al. (40) found that better glycemic control was significantly correlated with impulse control, in which inhibition plays a significant role. Therefore, poor inhibition increases the possibility of impulsive behavior, in turn leading to poor management of T1DM. It is important to become aware of the vicious cycle that arises when considering the effect of T1DM on inhibition and in turn poor inhibition on behavioral management, which ultimately, again, results in hyperglycemia, for example.

#### Subtheme 2.2: Poor behavioral and emotional control

The subtheme behavioral control is defined as the ability to inhibit, regulate, control, or perform certain behaviors that may influence T1DM management. Within the findings of this review, the scientific literature indicates that inhibition is strongly correlated with behavior regulation (39). Miller et al. (22) report that behavior regulation was a significant predictor of the quality of self-management behavior. (22, p. 23) conclude by highlighting the importance of moderating behavior: “… thus should be included in the final conditional model investigating how changes in behavioral regulation influence changes in diabetes self-management.” Hanna et al. (40) highlight the importance of behavior with regard to daily routine. Specific behaviors that have been associated with high inhibition and the adequate management of T1DM are seen in Duke et al. (20), who highlight the following: (1) not guessing doses; (2) not leaving home without sufficient goods and supplies; (3) not skipping glycemia checks; (4) good meal planning; and (5) making good choices in accordance with their treatment plan. Poor inhibition may as a result lead to an inability to perform, or a neglect of, the above-mentioned behaviors, in turn resulting in poor management of T1DM. Duke et al. (20) and Hamburger et al (39) support this finding, in that inhibition facilitates behavioral management and is a necessary component of treatment adherence. Treatment and management plan adherence is also a significant factor that has been correlated with behavioral control that is most prominently affected by inhibition.

Related to adherence in T1DM are the findings of Hamburger et al (39), who states that emotional control plays a significant role in the management of diabetes. Łuczyński et al. (41) report that the management of diabetes is influenced by one specific psychosocial factor, i.e., emotional control. Therefore, the ability to manage and control one’s emotions seems to contribute to the ability of an individual living with T1DM to exercise adequate management of the condition.

Inhibition has also been correlated with the ability to control emotions; for example, Caruso et al. (19) found that inhibition was significantly related to the emotional subscales in their study. This is of considerable relevance when one considers that in theme 1 it was reported that the limbic system is affected by fluctuations in glycemia. As a result, T1DM may affect an individual’s ability to utilize inhibition to control emotions, resulting in poor self-management.

## Implications of this research study

**Figure 2:**
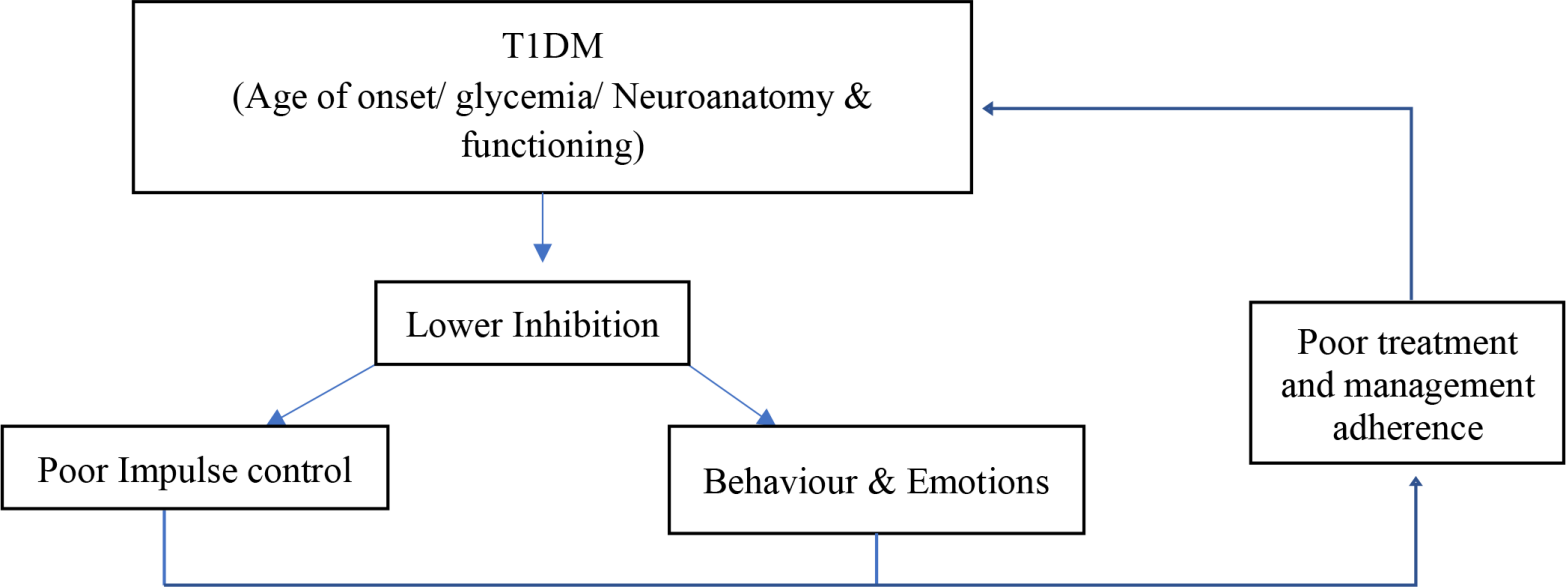
Cycle of interaction between T1DM, inhibition, and management.

The findings of this study indicate that inhibition plays a role in the behavior management of individuals living with T1DM. These findings most importantly highlight a vicious cycle that occurs between T1DM, inhibition, and the management of T1DM. First and foremost, T1DM has an effect on inhibition and its performance. It was found that inhibition may be affected by several factors associated with T1DM, such as hypo- or hyperglycemia and the individual’s age at the onset of disease. These factors or influences affect the neurodevelopment and functioning of an individual living with T1DM, thereby lowering the ability of an individual to utilize inhibition. In turn, as seen in the second theme, poor inhibition may result in poor adherence to treatment plans for, and self-management of, T1DM. Individuals living with T1DM may find it difficult to control their behaviors, impulses, and emotions and, therefore, to undergo treatment and achieve desired health outcomes. When one considers the fact that poor management and adherence behaviors may result in fluctuating glycemia, seizures, and neurological changes, one realizes that these factors may also affect inhibition performance. **Figure 2** depicts this cycle that may occur in individuals living with T1DM. In essence, T1DM affects inhibition, which in turn results in poor management and adherence, which in turn leads to lowered inhibition. The implication of this research study pertains specifically to a clear need for future research. This study thus provides the foundation for future researchers to identify the relationship more specifically between these three constructs.

## Limitations and recommendations

The limitations of this research study relate first to the limited and scarce literature available for review and analysis. The amount of scientific literature identified was greater than expected; however, exploration and discussion of inhibition were limited. Therefore, the findings of this study cannot accurately, or with conviction, assert that a definitive relationship exists between these three constructs (T1DM, inhibition, and behavior management). Despite this, the significance of this research study remains, as it raises a need for further exploration and investigation. Furthermore, the scientific literature identified did not consider the three constructs in the context of a relationship. The limited literature thus affects the trustworthiness of the findings. Another limitation of this study is that there is no scientific literature included in a South African context. Therefore, it is difficult to translate the findings of this review to a national context. This study does, however, highlight the need for the exploration of this relationship in a South African context.

Furthermore, the wide age range of participants in the articles included in the review is a strength, as it allowed for findings to arise regarding the role played by age at onset of T1DM on inhibition performance.

It is recommended that future research focuses specifically on inhibition in individuals living with T1DM. It is also recommended that future research considers the relationship between T1DM, inhibition, and behavior management, as the findings of the present study indicate that such a relationship may exist. Furthermore, one of the studies included developed an intervention focusing on behavioral management and T1DM, resulting in increased inhibition. Therefore, future researchers, in a national and international context, may consider developing and exploring possible interventions that can be applied to individuals living with T1DM.

## Conclusion

T1DM is a severe and chronic condition that disrupts the lives of affected individuals and their families. Often individuals may experience hypo- or hyperglycemia as a result, both of which severely affect the functioning of the individual and may lead to negative health outcomes. The consequences of T1DM have been correlated with poor cognitive functioning and neuroanatomical changes because of the fluctuating glycemia. One of the aspects that have not been thoroughly investigated, and for which research is sparse, is inhibition. Inhibition may play a significant role in T1DM, as it plays a significant role in behavioral management. The findings of the present study indicate that inhibition does play a role in T1DM. More specifically, it was found that inhibition is affected by T1DM, and that this results in poor adherence and management of the condition. In conclusion, it is vital to consider that a vicious cycle may be present between T1DM, inhibition, and behavior management, which may in turn result in negative health outcomes in individuals with T1DM.

## Author contributions

NR was responsible for the development and execution of the research study. This included the data gathering and analysis, and writing of the manuscript. ED was responsible for the development and monitoring of the research study. This included data gathering, data analysis, and the writing and editing of the manuscript. ED provided key insights into T1DM. KB assisted in the conceptualization and development of the research study. KB played a vital role in the monitoring and editing of the manuscript, and provided key insight into executive functioning. All authors contributed to the article and approved the submitted version.

## References

[1] Bade–White PA Obrzut JE. The neurocognitive effects of type 1 diabetes mellitus in children and young adults with and without hypoglycemia. J Dev Phys Disabil (2009) 21(5):425–40. doi: 10.1007/s10882-009-9151-y

[2] Goldberg A Scharf M Wiseman H. Sense of coherence and parenting representation among parents of adolescents with type 1 diabetes. J Pediatr Nurs (2017) 35:3–7. doi: 10.1016/j.pedn.2017.01.011 28728765

[3] Gregory JM Moore DJ Simmons JH. Type 1 diabetes mellitus. Pediatr Rev (2013) 34(5):203–15. doi: 10.1542/pir.34.5.203 23637249

[4] Kahanovitz L Sluss PM Russell SJ. Type 1 diabetes–a clinical perspective. Point Care (2017) 16(1):37. doi: 10.1097/POC.0000000000000125 28943810 PMC5606981

[5] Atkinson MA Eisenbarth GS Michels AW. Type 1 diabetes. Lancet (2014) 383(9911):69–82. doi: 10.1016/S0140-6736(13)60591-7 23890997 PMC4380133

[6] Chiang JL Maahs DM Garvey KC Hood KK Laffel LM Weinzimer SA. Type 1 diabetes in children and adolescents: a position statement by the American diabetes association. Diabetes Care (2018) 41(9):2026–44. doi: 10.2337/dci18-0023 PMC6105320 30093549

[7] Ferguson SC Blane A Perros P McCrimmon RJ Best JJ Wardlaw J. Cognitive ability and brain structure in type 1 diabetes: relation to microangiopathy and preceding severe hypoglycemia. Diabetes (2003) 52(1):149–56. doi: 10.2337/diabetes.52.1.149 12502506

[8] Kodl CT Seaquist ER. Cognitive dysfunction and diabetes mellitus. Endocrine Rev (2008) 29(4):494–511. doi: 10.1210/er.2007-0034 18436709 PMC2528851

[9] Northam EA Rankins D Cameron FJ. Therapy insight: The impact of type 1 diabetes on brain development and function. Nat. Clin Pract Neurol (2006) 2(2):78–86. doi: 10.1038/ncpneuro0097 16932529

[10] Suchy Y Butner J Wiebe DJ Campbell M Turner SL Berg CA. Executive cognitive functions and behavioral control differentially predict HbA1c in type 1 diabetes across emerging adulthood. J Int Neuropsychol Soc (2020) 26(4):353. doi: 10.1017/S1355617719001310 31822304 PMC7124994

[11] Todd JA. Etiology of type 1 diabetes. Immunity (2010) 32(4):457–67. doi: 10.1016/j.immuni.2010.04.001 20412756

[12] Wessels AM Scheltens P Barkhof F Heine RJ. Hyperglycaemia as a determinant of cognitive decline in patients with type 1 diabetes. Eur J Pharmacol (2008) 585(1):88–96. doi: 10.1016/j.ejphar.2007.11.080 18396273

[13] Broadley MM White MJ Andrew B. A systematic review and meta–analysis of executive function performance in type 1 diabetes mellitus. Psychosomatic Med (2017) 79(6):684–96. doi: 10.1097/PSY.0000000000000460 28207612

[14] Foland–Ross LC Buckingam B Mauras N Arbelaez AM Tamborlane WV Tsalikian E. Executive task–based brain function in children with type 1 diabetes: An observational study. PloS Med (2019) 16(12):e1002979. doi: 10.1371/journal.pmed.1002979 31815939 PMC6901178

[15] Moheet A Mangia S Seaquist ER. Impact of diabetes on cognitive function and brain structure. Ann New York Acad Sci (2015) 1353(60):60–71. doi: 10.1111/nyas.12807 26132277 PMC4837888

[16] Perantie DC Wu J Koller JM Lim A Warren SL Black KJ. Regional brain volume differences associated with hyperglycemia and severe hypoglycemia in youth with type 1 diabetes. Diabetes Care (2008) 30(9):2331–7. doi: 10.2337/dc07-0351 17575089

[17] American Diabetes Association. Diagnosis and classification of diabetes mellitus (2014). Available at: https://care.diabetesjournals.org/content/37/Supplement_1/S81.

[18] Brands AM Biessels GJ De Haan EH Kappelle LJ Kessels RP. The effects of type 1 diabetes on cognitive performance: a meta–analysis. Diabetes Care (2005) 28(3):726–35. doi: 10.2337/diacare.28.3.726 15735218

[19] Caruso NC Radovanovic B Kennedy JD Couper J Kohler M Kavanagh PS. Sleep, executive functioning and behaviour in children and adolescents with type 1 diabetes. Sleep Med (2014) 15(12):1490–9. doi: 10.1016/j.sleep.2014.08.011 25441750

[20] Duke DC Raymond JK Harris MA. The diabetes related executive functioning scale (DREFS): pilot results. Children's Health Care (2014) 43(4):327–44. doi: 10.1080/02739615.2013.870040

[21] Goethals ER Volkening LK Laffel LM. Executive dysfunction is associated with poorer health–related quality of life in adolescents with type 1 diabetes: Differences by sex. Qual. Life Res. (2021) 30(3):751–8. doi: 10.1007/s11136-020-02681-5 PMC8988089 33106962

[22] Miller MM Rohan JM Delamater A Shroff–Pendley J Dolan LM Reeves G. Changes in executive functioning and self–management in adolescents with type 1 diabetes: A growth curve analysis. J Pediatr Psychol (2013) 38(1):18–29. doi: 10.1093/jpepsy/jss100 23027720 PMC3695638

[23] Munshi M Grande L Hayes M Ayres D Suhl E Capelson R. Cognitive dysfunction is associated with poor diabetes control in older adults. Diabetes Care (2006) 29(8):1794–9. doi: 10.2337/dc06-0506 PMC1615865 16873782

[24] Ojo O Brooke J. Evaluating the association between diabetes, cognitive decline and dementia. Int J Environ Res Public Health (2015) 12(7):8281–94. doi: 10.3390/ijerph120708281 PMC4515722 26193295

[25] Ryan CM Williams TM. Effects of insulin–dependent diabetes on learning and memory efficiency in adults. J Clin and Experimental Neuropsychol (1993) 15(5):685–700. doi: 10.1080/01688639308402589 8276929

[26] Sachon C Grimaldi A Digy JP Pillon B Dubois B Thervet F. Cognitive function, insulin-dependent diabetes and hypoglycaemia. J Internal Med (1992) 231(5):471–5. doi: 10.1111/j.1365-2796.1992.tb00962.x 1602284

[27] Wredling R Levander S Adamson U Lins PE. Permanent neuropsychological impairment after recurrent episodes of severe hypoglycaemia in man. Diabetologia (1990) 33(3):152–7. doi: 10.1007/BF00404042 2184066

[28] Goldstein S Naglieri JA Dana Princiotta D Otero TM. Introduction: A history of executive functioning as a theoretical and clinical construct. In: Goldstein S Naglieri JA, editors. Handbook of executive functioning. Springer, New York (2014). p. 3–13.

[29] Daucourt MC Schatschneider C Connor CM Al Otaiba S Hart SA. Inhibition, updating working memory, and shifting predict reading disability symptoms in a hybrid model: Project KIDS. Front Psychol (2018) 9:238. doi: 10.3389/fpsyg.2018.00238 29662458 PMC5890166

[30] Chung HJ Weyandt LL Swentosky A. The physiology of executive functioning. In: Handbook of executive functioning. Springer, New York (2014).

[31] Goldman–Rakic PS Thierry AM Glowinski J Goldman–Rakic PS Christen Y. Motor and cognitive function of the prefrontal cortex. Springer Berlin (1994).

[32] Diamond A. Executive functions. Annu Rev Psychol (2013) 64:135–68. doi: 10.1146/annurev-psych-113011-143750 PMC4084861 23020641

[33] Swanson V Maltinsky W. Motivational and behaviour change approaches for improving diabetes management. Pract Diabetes (2019) 36(4):121–5. doi: 10.1002/pdi.2229

[34] Hood KK Hilliard M Piatt G Ievers-Landis CE. Effective strategies for encouraging behavior change in people with diabetes. Diabetes Management (London, England). 2015 5(6):499. 30100925 PMC6086609

[35] Stephani V Opoku D Beran D. Self–management of diabetes in Sub–Saharan Africa: a systematic review. BMC Public Health (2018) 18(1):1–11. doi: 10.1186/s12889-018-6050-0 PMC6162903 30268115

[36] Carnwell R Daly W. Strategies for the construction of a critical review of the literature. Nurse education in practice. (2001) 1(2):57–63. 10.1054/nepr.2001.0008 19036245

[37] Grant MJ Booth A. A typology of reviews: An analysis of 14 review types and associated methodologies. Health Inf Libraries J (2009) 26(2):91–108. doi: 10.1111/j.1471-1842.2009.00848.x 19490148

[38] Graveling AJ Deary IJ Frier BM. Acute hypoglycemia impairs executive cognitive function in adults with and without type 1 diabetes. Diabetes Care (2013) 36(10):3240–6. doi: 10.2337/dc13-0194 PMC3781527 23780950

[39] Hamburger ER Lyttle M Compas BE Jaser SS. Performance–based and questionnaire measures of executive function in adolescents with type 1 diabetes. J Behav Med (2019) 42(6):1041–9. doi: 10.1007/s10865-019-00027-2 PMC6746613 30879224

[40] Hanna KM Kupzyk KA Hansen JR Jones-Ryan ML Drincic AT. Association of habits, triggers, glycaemic control, routines, stress and impulse control among emerging adults with type 1 diabetes. Diabetic Med (2021) 38(3):e14370. doi: 10.1111/dme.14370 32745273

[41] Łuczyński W Łazarczyk I Szlachcikowska I Kiernozek Ż. Kaczmarek A Szylaj O. The empowerment of adolescents with type 1 diabetes is associated with their executive functions. BioMed Res In. (2019) 2019. doi: 10.1155/2019/5184682 PMC6515027 31183368

[42] Ohmann S Popow C Rami B König M Blaas S Fliri C. Cognitive functions and glycemic control in children and adolescents with type 1 diabetes. Psychol Med (2010) 40(1):95–103. doi: 10.1017/S0033291709005777 19400976

[43] Strachan MW Deary IJ Ewing FM Frier BM. Recovery of cognitive function and mood after severe hypoglycemia in adults with insulin–treated diabetes. Diabetes Care (2000) 23(3):305–12. doi: 10.2337/diacare.23.3.305 10868856

[44] Rovet J Alvarez M. Attentional functioning in children and adolescents with IDDM. Diabetes Care (1997) 20(5):803–10. doi: 10.2337/diacare.20.5.803 9135946

[45] Braun V Clarke V. Using thematic analysis in psychology. Qual Res Psychol (2006) 3:77–101. doi: 10.1191/1478088706qp063oa

[46] Maguire M Delahunt B. Doing a thematic analysis: A practical, step–by–step guide for learning and teaching scholars. All Ireland J Teach Learn Higher Educ (2017) 9(3):33501–33514.

[47] Marcovecchio ML Tossavainen. PH Dunger DB. Prevention and treatment of microvascular disease in childhood type 1 diabetes. Br Med Bull (2010) 94:145–64. doi: 10.1093/bmb/ldp053 20053672

[48] Povroznik JM Ozga JE Haar CV Engler-Chiurazzi EB. Executive (dys) function after stroke: special considerations for behavioral pharmacology. Behavioural pharmacology (2018) 29(7):638. 30215622 10.1097/FBP.0000000000000432 PMC6152929

[49] Bechara A Tranel D Damasio H. Characterization of the decision-making deficit of patients with ventromedial prefrontal cortex lesions. Brain (2000) 123(11):2189–202. 10.1093/brain/123.11.2189 11050020

[50] Binder LM. Emotional problems after stroke. Stroke (1984) 15(1):174–7. doi: 10.1161/01.str.15.1.174 6695423

[51] Poulin V Korner‐Bitensky N Dawson DR. Stroke‐specific executive function assessment: A literature review of performance‐based tools. Australian Occupational Therapy Journal 60(1):3–19. 10.1111/1440-1630.12024 23414185

[52] Blasi G Goldberg TE Weickert T Das S Kohn P Zoltick B. Brain regions underlying response inhibition and interference monitoring and suppression. Eur J Neurosci (2006) 23(6):1658–64. doi: 10.1111/j.1460-9568.2006.04680.x 16553630

[53] Arain M Haque M Johal L Mathur P Nel W Rais A. Maturation of the adolescent brain. Neuropsychiatr Dis Treat (2013) 9:449–61. doi: 10.2147/NDT.S39776 PMC3621648 23579318

[54] Morrongiello BA Lasenby–Lessard J. Psychological determinants of risk taking by children: an integrative model and implications for interventions. Injury Prev (2007) 13(1):20–5. doi: 10.1136/ip.2005.011296 PMC2610559 17296684

[55] Russo VC Higgins S Werther GA Cameron FJ. Effects of fluctuating glucose levels on neuronal cells *in vitro* . Neurochemical Res (2012) 37(8):1768–82. doi: 10.1007/s11064-012-0789-y 22565596

[56] Kern S Oakes TR Stone CK McAuliff EM Kirschbaum C Davidson RJ. Glucose metabolic changes in the prefrontal cortex are associated with HPA axis response to a psychosocial stressor. Psychoneuroendocrinology (2008) 33(4):517–29. doi: 10.1016/j.psyneuen.2008.01.010 PMC2601562 18337016

[57] Page GLJ Laight D Cummings MH. Thiamine deficiency in diabetes mellitus and the impact of thiamine replacement on glucose metabolism and vascular disease. Int Clin Pract (2011) 65(6):684–90. doi: 10.1111/j.1742-1241.2011.02680.x 21564442

[58] Mortby ME Janke AL Anstey KJ Sachdev PS Cherbuin N. High “normal” blood glucose is associated with decreased brain volume and cognitive performance in the 60s: The PATH through life study. PloS One (2013) 8(9):e73697. doi: 10.1371/journal.pone.0073697 24023897 PMC3762736

[59] Rosenthal JM Amiel SA Yágüez L Bullmore E Hopkins D Evans M. The effect of acute hypoglycemia on brain function and activation: a functional magnetic resonance imaging study. Diabetes (2001) 50(7):1618–26. doi: 10.2337/diabetes.50.7.1618 11423484

[60] Liu Q Zhu X Ziegler A Shi J. The effects of inhibitory control training for preschoolers on reasoning ability and neural activity. Sci Rep (2015) 5(1):1–11. doi: 10.1038/srep14200 PMC4585799 26395158

[61] Dillon DG Pizzagalli DA. Inhibition of action, thought, and emotion: a selective neurobiological review. Appl Prev Psychol (2007) 12(3):99–114. doi: 10.1016/j.appsy.2007.09.004 19050749 PMC2396584

[62] Stupiansky NW Hanna KM Slaven JE Weaver MT Fortenberry JD. Impulse control, diabetes-specific self-efficacy, and diabetes management among emerging adults with type 1 diabetes. J Pediatr Psychol (2013) 38(3):247–54. doi: 10.1093/jpepsy/jss110 PMC3604821 23115219

[63] Bari A Robbins TW. Inhibition and impulsivity: behavioral and neural basis of response control. Prog Neurobiol (2013) 108:44–79. doi: 10.1016/j.pneurobio.2013.06.005 23856628

[64] Hammond CJ Potenza MN Mayes LC. Development of impulse control, inhibition, and self–regulatory behaviors in normative populations across the lifespan. In: Grant JE Potenza MN, editors. The Oxford handbook of impulse control disorders. Oxford University Press, New York (2012). p. 232–42.

[65] Roberts W Fillmore MT Milich R. Linking impulsivity and inhibitory control using manual and oculomotor response inhibition tasks. Acta Psychologica (2011) 138(3):419–28. doi: 10.1016/j.actpsy.2011.09.002 PMC3205291 21982865

